# The role of thromboelastography and conventional coagulation tests in missed and recurrent spontaneous abortion: a subgroup analysis based on embryonic chromosome status

**DOI:** 10.3389/fendo.2026.1787627

**Published:** 2026-07-15

**Authors:** Yanqing Wu, Min Li, Yaqi Yan, Fangshu Le, Xin Du, Xiaoling Tao

**Affiliations:** Gynaecology Department, Maternal and Child Health Hospital of Hubei Province, Tongji Medical College, Huazhong University of Science and Technology, Wuhan, China

**Keywords:** activated partial thromboplastin time (aPTT), coagulation dysregulation, missed abortion, recurrent spontaneous abortion (RSA), R-time

## Abstract

**Background:**

The contribution of coagulation parameters to pregnancy loss, especially the TEG, independent of embryonic chromosomal anomalies, is not well defined. This study investigated the association of coagulation profiles with Missed Abortion and Recurrent Spontaneous Abortion (RSA), accounting for chromosomal status.

**Methods:**

In this cross-sectional study, 648 participants were enrolled (398 missed abortion cases, 250 controls). From the 398 cases, 94 patients with recurrent spontaneous abortion (RSA) were selected and compared with healthy controls who had a normal current pregnancy and no history of adverse pregnancy outcomes (n = 233). Preoperative assessments included conventional coagulation tests, thromboelastography (TEG), and relevant clinical covariates (e.g., gravidity, parity, and history of adverse pregnancies). Postoperative embryonic chromosomal status was determined by CNV-seq. Multivariable logistic regression models adjusting for potential confounders were used to evaluate the associations.

**Results:**

Prolonged APTT and R-time were significantly associated with increased risks of missed abortion (APTT: OR = 1.14, 95% CI: 1.08-1.21; R-time: OR = 1.85, 95% CI: 1.38-2.49) and RSA (APTT: OR = 1.20, 95% CI: 1.09-1.33; R-time: OR = 1.81, 95% CI: 1.37-2.38). These associations persisted after stratifying by chromosomal normality in missed abortion. While, APTT and R-time values did not differ significantly in missed abortion between CNV-seq Negative and Positive groups.

**Conclusion:**

Prolonged APTT and R-time are independently associated with missed abortion and RSA, suggesting a role for impaired coagulation initiation in pregnancy loss etiology. These parameters may serve as useful clinical biomarkers for prognostic evaluation of pregnancy loss.

## Background

Recurrent spontaneous abortion (RSA) affects approximately 1-5% of reproductive-age couples and remains unexplained in 50-75% of cases despite comprehensive investigation (1). Both sporadic and recurrent miscarriage carry significant psychosocial consequences, including increased risks of anxiety, depression, and post-traumatic stress disorder. Irrespective of etiology, RSA confers a threefold higher odds of moderate-to-severe antenatal depression and a two-fold increase in post-traumatic stress disorder relative to single miscarriage, with psychopathology persisting >12 months in both women and their partners (2). The condition imposes substantial clinical, emotional, and socioeconomic burdens on affected families and healthcare systems, representing a leading cause of pregnancy-related morbidity.

The etiology of recurrent or missed miscarriage is multifactorial, encompassing embryonic/parental genetic abnormalities, uterine anatomical defects, endocrine dysfunctions, and immune-mediated disorders (3). Even after standard clinical evaluation, approximately 15% of cases are classified as “unexplained”, highlighting a significant knowledge gap (4). Embryonic aneuploidy accounts for a substantial proportion of early losses (approximately 29-60% in RSA cohorts; >50% of early losses overall) (5). Uterine anomalies occur in 10-13% of RSA patients, while endocrine factors include polycystic ovarian syndrome (PCOS), thyroid disorders, hyperprolactinemia, and obesity (6, 7).

Among acquired prothrombotic states, antiphospholipid syndrome (APS) is a well-established cause of obstetric complications, though the significance of other thrombophilia’s and empirical anticoagulation in unexplained RSA remains controversial (8). The causal relationship between hypercoagulability and miscarriage continues to be debated, with meta-analyses reporting inconsistent results for common thrombophilic mutations such as factor V Leiden and prothrombin gene mutations (9–11).

APS represents a well-documented autoimmune disorder consistently associated with late pregnancy loss, primarily mediated through antiphospholipid antibodies (aPL) that increase thrombosis risk and various obstetric complications. Lupus anticoagulant, a type of aPL, demonstrates a significant association with late fetal loss (OR 3.47) (12). Additionally, aPS/PT antibodies have been associated with adverse pregnancy outcomes, showing odds ratios of 5.96 for early miscarriage and 7.28 for fetal loss in healthy women with unexplained RSA (13). However, physiological hypercoagulability during pregnancy complicates distinguishing pathological from benign states, with one ROTEM-based study finding no significant coagulation differences between women with threatened miscarriage and healthy controls (14).

The activated partial thromboplastin time (APTT) assesses the intrinsic and common coagulation pathways, sensitive to deficiencies or inhibitions of factors VIII, IX, XI, and XII. Shortened APTT and elevated factor VIII levels have correlated with recurrent pregnancy loss, with abbreviated APTT emerging as an independent RSA risk factor (15). Thromboelastography (TEG) combined with antithrombin III and D-dimer assays shows promising predictive value for RSA occurrence, with studies demonstrating coagulation-anticoagulation imbalances characterized by altered TEG parameters in RSA patients compared to those with normal reproductive histories (16–18). Although some studies suggest that TEG parameters indicate hypercoagulability in unexplained RSA, others find no significant evidence of such a state (19).

Despite growing evidence linking coagulation abnormalities to adverse pregnancy outcomes, the distinct roles of global coagulation parameters—particularly TEG indices and conventional markers—across different miscarriage subtypes remain poorly delineated. Existing studies have predominantly focused broadly on RSA or missed abortion without accounting for genetic etiologies or stratifying by critical confounders such as gestational age, BMI, or cytogenetic normality. A notable research gap exists in comparative analyses between cytogenetically normal and abnormal losses, essential for determining whether coagulation dysfunction operates independently of chromosomal anomalies. Therefore, this study aims to comprehensively investigate coagulation profiles in RSA, missed abortion, and CNV-seq-stratified cohorts, utilizing multivariate adjustment and subgroup analyses to identify specific and independent associations.

## Materials and methods

### Study design and participants

This cross-sectional study utilized data from patients presenting at the Department of Gynecology, Hubei Maternal and Child Health Hospital, between March 1, 2024, and December 31, 2024. The study protocol adhered to the ethical principles of the Declaration of Helsinki and received approval from the Institutional Ethics Committee (Approval No.: 2024-101-01).

The case group comprised 398 women with gestational ages <20 weeks diagnosed with missed abortion. Diagnoses were confirmed by attending gynecologists (associate chief physician or higher) based on clinical criteria from the 9th edition of Obstetrics and Gynecology. The definition of RSA is not universally standardized. The American Society for Reproductive Medicine (ASRM) traditionally defines RSA as two or more failed clinical pregnancies, whereas the European Society of Human Reproduction and Embryology (ESHRE) and the Royal College of Obstetricians and Gynaecologists (RCOG) use a threshold of three or more consecutive losses. In this study, we adopted the definition of two or more consecutive pregnancy losses for the following reasons: (1) our clinical practice follows the ASRM guideline; (2) earlier identification of RSA allows for timely intervention; (3) this definition is consistent with several previous studies on coagulation profiles in RSA, facilitating comparison of our findings with the literature; and (4) given the sample size of our missed abortion cohort, using the ≥2 definition provided a sufficient number of RSA patients for meaningful subgroup analysis. The control group included 250 healthy pregnant women who were before 20 weeks of gestation and requested voluntary termination of pregnancy during the same period. RSA is defined as two or more consecutive pregnancy losses before 28 weeks of gestation, including consecutive biochemical pregnancies, and its etiological spectrum is closely correlated with the gestational age at miscarriage. The RSA subgroup was identified from the missed abortion cohort based on a history of ≥1 prior pregnancy loss (in addition to the current missed abortion). Hence, the RSA analysis represents a subgroup analysis nested within the missed abortion analysis. From the 398 patients with missed abortion, 94 patients who met the criteria for RSA were selected for subsequent analysis. All controls had confirmed intrauterine pregnancies with detectable fetal cardiac activity by ultrasound. Inclusion criteria for the case group were: age 18–45 years; meeting clinical diagnostic criteria for missed abortion; no antiphospholipid antibody syndrome diagnosis; no heparin use during current pregnancy; no personal thrombosis history; and no chronic systemic diseases (e.g., hypertension, diabetes). Control group inclusion criteria were: age 18–45 years; confirmed viable intrauterine pregnancy by ultrasound; absence of chronic systemic diseases. Exclusion criteria for both groups included: known genetic disorders or reproductive tract structural abnormalities; history of chromosomal abnormalities in either partner; history of active infections (e.g., TORCH); and long-term smoking or alcohol abuse. After applying these criteria, 648 participants were included in the final analysis. Comprehensive clinical data were collected within 1–2 days pre-surgery, including demographic information, anthropometric measurements, and biochemical profiles.

### Blood sampling and coagulation assays

Peripheral venous blood (3 mL) was collected via venipuncture into vacuum tubes containing 0.109 mol/L sodium citrate anticoagulant (anticoagulant:blood ratio 1:9). Samples were centrifuged at 3000 rpm for 10 minutes to obtain platelet-poor plasma. Conventional coagulation parameters—prothrombin time (PT), activated partial thromboplastin time (APTT), thrombin time (TT), fibrinogen (FIB), D-dimer, and International normalized Ratio (INR) —were measured using a calibrated automated coagulation analyzer (STAGO STA-R Evolution, France; reagents: STA^®^-PTT Automate 5). Global viscoelastic hemostatic properties were assessed by thromboelastography (TEG) using the TEG^®^ 5000 Hemostasis Analyzer (Haemonetics Corporation, USA) with corresponding reagents (Kaolin activator, Haemonetics) and software per manufacturer instructions. The following parameters were quantitatively evaluated: reaction time (R-time), kinetics of clot formation (K), α-angle, maximum amplitude (MA), lysis at 30 minutes (LY30), Estimated Percent Lysis (EPL) and Clotting index (CI). All assays were performed by technicians blinded to the clinical status of participants. Intra-assay and inter-assay coefficients of variation were <5% for all measured parameters.

### CNV-seq analysis

Following surgical abortion, villi were collected from the chorionic sac and rinsed with normal saline to minimize maternal cell contamination. Aneuploidy and copy number variation (CNV) detection was performed as previously described (20), utilizing low-coverage whole-genome sequencing capable of identifying aneuploidies, unbalanced structural rearrangements, and CNVs >100 Kb.

Briefly, genomic DNA was extracted using the Amp Genomic DNA Kit (TIANGEN, China) per manufacturer protocol. Subsequently, 2.5 ng of fragmented genomic DNA was used to construct sequencing libraries. Purified libraries were sequenced on the NextSeq 550AR platform (Illumina, USA). Uniquely aligned reads were counted per 100 Kb window and averaged along each chromosome. Sequencing data were aligned to the human reference genome, with bioinformatic analyses conducted to identify chromosomal abnormalities.

Large CNVs (≥10 Mb) were defined as segmental aneuploidies, while sub-microscopic CNVs (<10 Mb) were classified as microdeletions/duplications. Identified CNVs were annotated and interpreted using genomic and phenotype databases (UCSC Genome Browser, ClinGene, OMIM, DECIPHER, DGV, PubMed). A quantitative, evidence-based scoring framework adhering to ACMG technical standards was applied to reassess CNV pathogenicity. Benign CNVs were regarded as normal chromosomal variants (21).

### Statistical analysis

All analyses were conducted using R statistical software (version 3.3.2) and Free Statistics software (version 2.2.0). Statistical significance was set at two-sided p<0.05. Continuous variables are presented as means ± standard deviations or medians with interquartile ranges. Differences were assessed using one-way ANOVA (normally distributed data) or Kruskal-Wallis test (skewed distributions).

Binary logistic regression models evaluated associations between coagulation parameters and outcomes. Three models were developed: Model 1 (unadjusted); Model 2 (adjusted for age and BMI); Model 3 (adjusted for age, BMI, and days of amenorrhea). For multiple comparisons, Bonferroni correction was applied where appropriate.

## Results

### Characteristics of the subjects included normal and missed abortion

From the **Table 1**, we found the mean age of participants was similar between normal (31.8 ± 6.2 years) and missed abortion (32.3 ± 4.6 years, *P* =0.213) groups. Days of amenorrhea showed no significant difference (66.2 ± 24.3 vs. 66.7 ± 12.6 days, *P*=0.736). D-dimer levels were comparable (0.3 [0.2,0.5] vs. 0.3 [0.2,0.4] mg/L, p=0.4), with no significant differences in TT, PT, FIB, or INR (all *P*>0.05). APTT was significantly prolonged in the missed abortion group (30.5 ± 3.5 vs. 29.4 ± 2.6 s, *P <*0.001). Clotting index (CI), Estimated Percent Lysis (EPL), and lysis at 30 minutes (LY30) were significantly higher in the normal group (*P* = 0.03, *P*=0.004, and *P*=0.019, respectively). No significant differences were observed in MA, angle, or K parameters. R-time was significantly higher in the missed abortion group (5.1 ± 1.0 vs. 4.9 ± 0.9 min, *P*=0.001).

**Table 1:** Coagulation status in women with normal pregnancy and missed abortion.

Characteristics	Normal (n = 250)	Patients with missed abortion (n = 398)	*P*-valu*e*
Age (years)	31.8 ± 6.2	32.3 ± 4.6	0.213
BMI (kg/m²)	22.1 ± 3.2	22.7 ± 3.6	0.018
Days of amenorrhea	66.2 ± 24.3	66.7 ± 12.6	0.736
Num_of_Parity	0.6 ± 0.8	0.3 ± 0.6	< 0.001
Num_abortion	1.0 (0.0, 2.0)	1.0 (0.0, 1.0)	0.005
Num Spontaneous abortion	0.0 ± 0.2	0.1 ± 0.3	0.138
Num miss abortion	0.0 ± 0.2	0.3 ± 0.6	< 0.001
Num induced abortion	1.0 (0.0, 2.0)	0.0 (0.0, 1.0)	< 0.001
D-dimer(mg/L)	0.3 (0.2, 0.5)	0.3 (0.2, 0.4)	0.4
TT(s)	13.7 ± 1.2	13.8 ± 1.4	0.866
PT(s)	11.4 ± 0.9	11.5 ± 1.4	0.724
APTT(s)	29.4 ± 2.6	30.5 ± 3.5	< 0.001
FIB(g/L)	3.1 ± 0.8	3.1 ± 1.9	0.949
INR	1.1 ± 0.7	1.1 ± 0.1	0.466
CI	1.3 (0.7, 2.2)	1.1 (0.3, 1.9)	0.03
EPL (%)	0.0 (0.0, 1.0)	0.0 (0.0, 0.6)	0.004
LY30 (%)	0.0 (0.0, 0.1)	0.0 (0.0, 0.0)	0.019
MA (mm)	62.9 ± 4.3	62.2 ± 7.7	0.175
Angle(degrees)	67.3 (64.1, 69.5)	66.9 (63.9, 69.6)	0.55
K(min)	1.7 ± 0.9	1.6 ± 0.6	0.767
R-time(min)	4.9 ± 0.9	5.1 ± 1.0	0.001

Data presented as mean ± standard deviation or median (interquartile range) as appropriate. K, Kinetics of clot formation, α-angle; MA, maximum amplitude; LY30, lysis at 30 minutes; EPL, Estimated Percent Lysis; CI, Clotting index. *P* value < 0.05 was considered statistically significant. The specific test used for each variable (e.g., age, gestational age) is indicated in the corresponding row.

### Association of APTT and R-time with missed abortion

Logistic regression analyses demonstrated significant associations between both APTT and R-time with missed abortion across all models (**Table 2**). In unadjusted Model 1, APTT showed an OR of 1.12 (95% CI: 1.06-1.19, *P*<0.001) per unit increase, while R-time demonstrated an OR of 1.82 (95% CI: 1.38-2.40, *P*<0.001). After adjusting for age and BMI (Model 2), associations remained robust (APTT OR = 1.14 [1.07-1.21], R-time OR = 1.81 [1.37-2.38]). Further adjustment for days of amenorrhea (Model 3) yielded similar results (APTT OR = 1.14 [1.08-1.21], R-time OR = 1.85 [1.38-2.49]).

**Table 2:** Odds ratios and 95% confidence intervals for the association of APTT and R-time with missed abortion.

Parameter	Model 1		Model 2		Model 3	
	OR (95%CI)	*P*-Value	OR (95%CI)	*P*-Value	OR (95%CI)	*P*-Value
APTT (s)	1.12 (1.06~1.19)	<0.001	1.14 (1.07~1.21)	<0.001	1.14 (1.08~1.21)	<0.001
R-time	1.82 (1.38~2.4)	<0.001	1.81 (1.37~2.38)	<0.001	1.85 (1.38~2.49)	<0.001

Model 1: Unadjusted; Model 2: Adjusted for age and BMI; Model 3: Adjusted for age, BMI, and days of amenorrhea. *P* value < 0.05 was considered statistically significant and *P*-value used for each variable is indicated in the corresponding row.

### Subgroup analyses for the association of APTT and R-time with missed abortion

**Figure 1:**
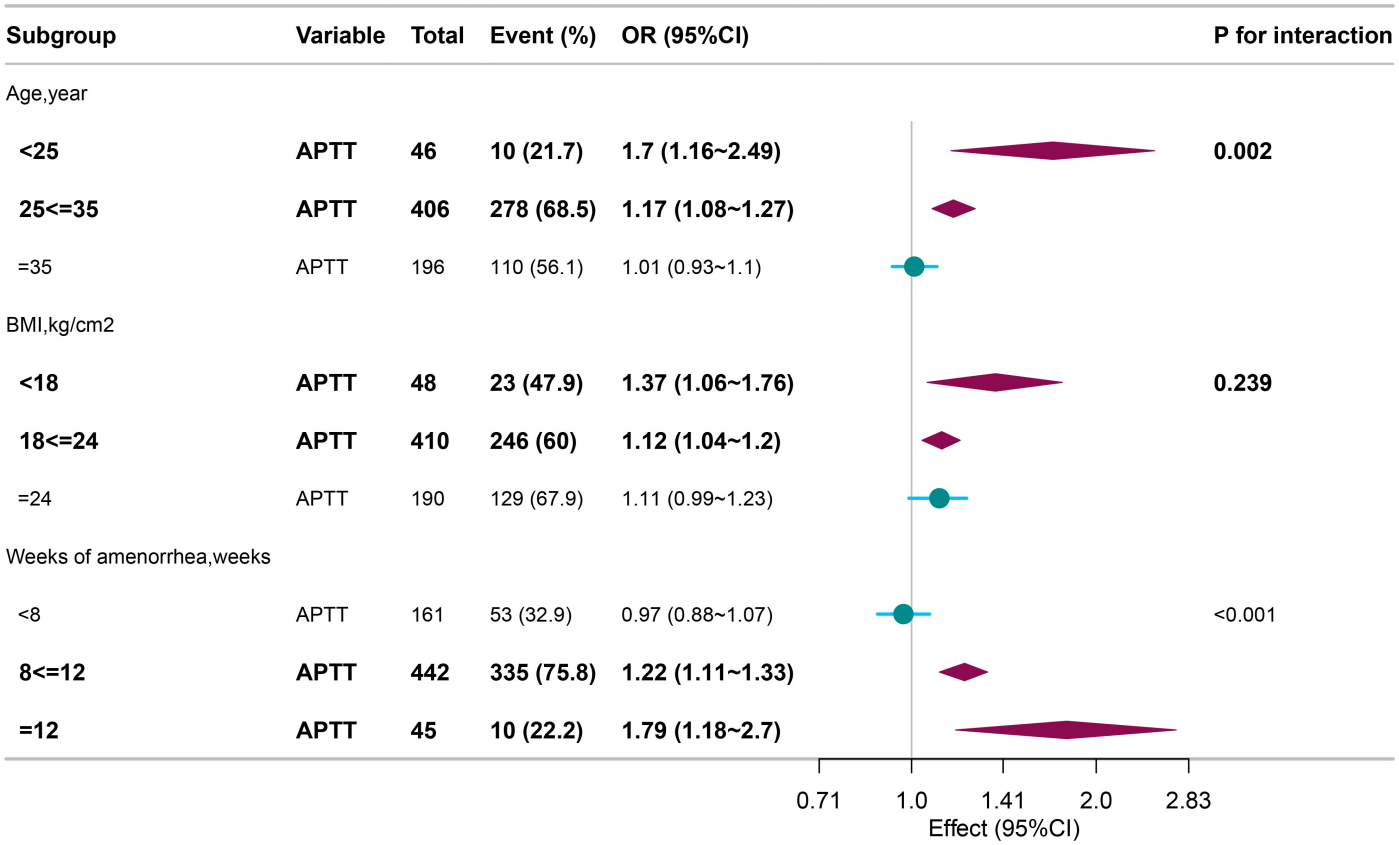
Forest plot showing the interaction effect of APTT with missed abortion across different subgroups.

**Figure 2:**
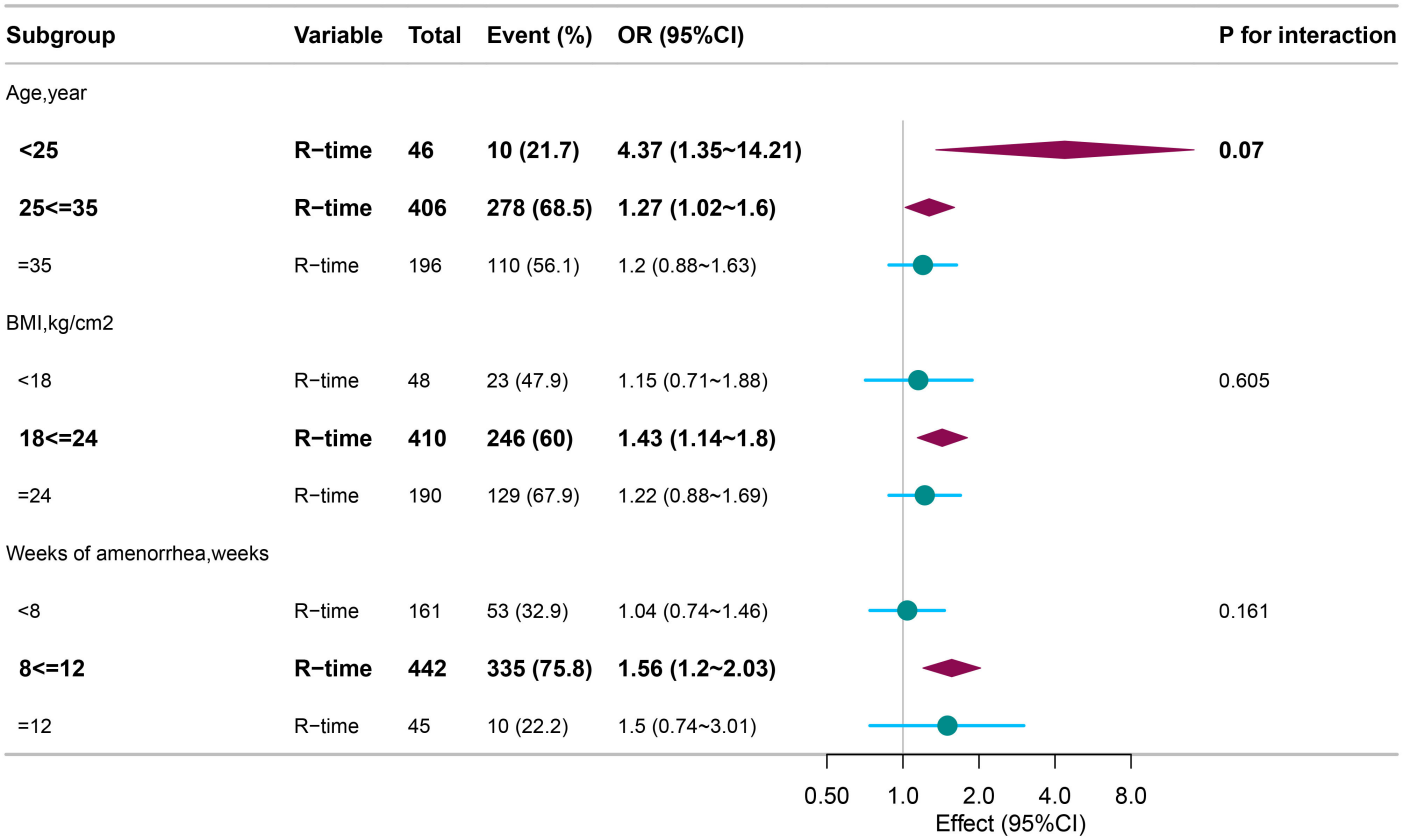
Forest plot showing the interaction effect of R with missed abortion across different subgroups.

Subgroup analyses revealed variations in associations across demographic and clinical categories (**Table 3**). Forest plots (**Figures 1**, **2**) illustrate these associations. The strongest associations for both parameters were observed in women <25 years (APTT OR = 1.70 [1.16-2.49]; R-time OR = 4.37 [1.35-14.21]). Significant associations were also found in the 25–35 years group (APTT OR = 1.17 [1.08-1.27]; R-time OR = 1.27 [1.02-1.60]), but not in those ≥35 years. BMI stratification showed significant associations in the 18–24 kg/m² group (APTT OR = 1.12 [1.04-1.20]; R-time OR = 1.43 [1.14-1.80]). For amenorrhea duration, the strongest associations emerged in the 8–12 weeks category (APTT OR = 1.22 [1.11-1.33]; R-time OR = 1.56 [1.20-2.03]).

**Table 3:** Subgroup analyses for the association of APTT and R-time with missed abortion.

Subgroup	Total	Event (%)	APTT	R-time
			OR (95%CI)	OR (95%CI)
Age(years)				
<25	46	10 (21.7)	1.7 (1.16~2.49)	4.37 (1.35~14.21)
25-35	406	278 (68.5)	1.17 (1.08~1.27)	1.27 (1.02~1.6)
≥35	196	110 (56.1)	1.01 (0.93~1.1)	1.2 (0.88~1.63)
BMI(kg/cm2)				
<18	48	23 (47.9)	1.37 (1.06~1.76)	1.15 (0.71~1.88)
18-24	410	246 (60)	1.12 (1.04~1.2)	1.43 (1.14~1.8)
≥24	190	129 (67.9)	1.11 (0.99~1.23)	1.22 (0.88~1.69)
Weeks of amenorrhea				
<8	161	53 (32.9)	0.97 (0.88~1.07)	1.04 (0.74~1.46)
8-12	442	335 (75.8)	1.22 (1.11~1.33)	1.56 (1.2~2.03)
≥12	45	10 (22.2)	1.79 (1.18~2.7)	1.5 (0.74~3.01)

### Characteristics of the subjects included control and RSA

Among 250 healthy pregnant women in early pregnancy who requested voluntary termination of pregnancy, there were 7 cases with a history of spontaneous miscarriage and 10 cases of missed miscarriage. For comparison with RSA, we selected 233 healthy individuals with no prior history of adverse pregnancy outcomes, which means the RSA findings are derived from a subset of the MA group. As showed in **Table 4**, the RSA group (n=94) was slightly older than controls (n=233) (33.3 ± 4.4 vs. 31.5 ± 6.2 years, *P*=0.015) and showed prolonged APTT (30.6 ± 3.5 vs. 29.4 ± 2.6 s, *P*<0.001) and R-time (5.4 ± 1.1 vs. 4.8 ± 0.8 min, *P*<0.001). The RSA group had lower CI (1.0 vs. 1.3, *P*=0.019), EPL (0.0 vs. 0.0, *P*=0.006), LY30 (0.0 vs. 0.0, *P*=0.004), and MA (60.6 ± 12.2 vs. 62.9 ± 4.3 mm, *P*=0.013). No significant differences were found in BMI, days of amenorrhea, D-dimer, TT, PT, FIB, INR, angle, or K value.

**Table 4:** Coagulation status in women with normal pregnancy and recurrent spontaneous abortion.

Characteristics	Control (n = 233)	RSA (n = 94)	*P*-valu*e*
Age (years)	31.5 ± 6.2	33.3 ± 4.4	0.015
BMI (kg/m²)	22.0 ± 3.3	22.8 ± 3.4	0.062
Days of amenorrhea	66.8 ± 24.6	63.4 ± 11.2	0.202
D-dimer(mg/L)	0.3 (0.2, 0.5)	0.3 (0.2, 0.4)	0.706
TT(s)	13.7 ± 1.3	14.0 ± 0.9	0.081
PT(s)	11.4 ± 0.9	11.5 ± 1.3	0.58
APTT(s)	29.4 ± 2.6	30.6 ± 3.5	< 0.001
FIB(g/L)	3.1 ± 0.7	3.0 ± 0.6	0.276
INR	1.1 ± 0.8	1.1 ± 0.1	0.732
CI	1.3 (0.7, 2.2)	1.0 (0.1, 1.9)	0.019
EPL (%)	0.0 (0.0, 1.0)	0.0 (0.0, 0.3)	0.006
LY30 (%)	0.0 (0.0, 0.1)	0.0 (0.0, 0.0)	0.004
MA (mm)	62.9 ± 4.3	60.6 ± 12.2	0.013
Angle(degrees)	67.5 (64.2, 69.5)	66.5 (64.0, 70.1)	0.496
K(min)	1.6 ± 0.9	1.6 ± 0.5	0.706
R-time(min)	4.8 ± 0.8	5.4 ± 1.1	< 0.001

Data presented as mean ± standard deviation or median (interquartile range) as appropriate. K, Kinetics of clot formation, α-angle; MA, maximum amplitude; LY30, lysis at 30 minutes; EPL, Estimated Percent Lysis; CI, Clotting index. *P* value < 0.05 was considered statistically significant and *P*-value used for each variable is indicated in the corresponding row.

### Association of APTT and R-time with RSA

Multivariable logistic regression analyses demonstrated that both prolonged APTT and R-time were significantly associated with increased RSA risk across all models (**Table 5**). In unadjusted Model 1, each unit increase in APTT was associated with an OR of 1.19 (95% CI: 1.09-1.31, *P*<0.001), and each unit increase in R-time with an OR of 1.81 (95% CI: 1.37-2.38, *P*<0.001). Adjustments for age and BMI (Model 2) and additional adjustment for days of amenorrhea (Model 3) yielded consistent results.

**Table 5:** Odds ratios and 95% confidence intervals for the association of APTT and R-time with recurrent spontaneous abortion.

Peremeter	Model 1		Model 2		Model 3	
	OR (95%CI)	*P*-Value	OR (95%CI)	*P*-Value	OR (95%CI)	*P*-Value
APTT (s)	1.19 (1.09~1.31)	<0.001	1.19 (1.09~1.31)	<0.001	1.2 (1.09~1.33)	<0.001
R-time	1.81 (1.37~2.38)	<0.001	1.8 (1.36~2.37)	<0.001	1.81 (1.37~2.38)	<0.001

Model 1: Unadjusted; Model 2: Adjusted for age and BMI; Model 3: Adjusted for age, BMI, and days of amenorrhea. *P* value < 0.05 was considered statistically significant and *P*-value used for each variable is indicated in the corresponding row.

### Subgroup analyses for the association of APTT and R-time with RSA

**Figure 3:**
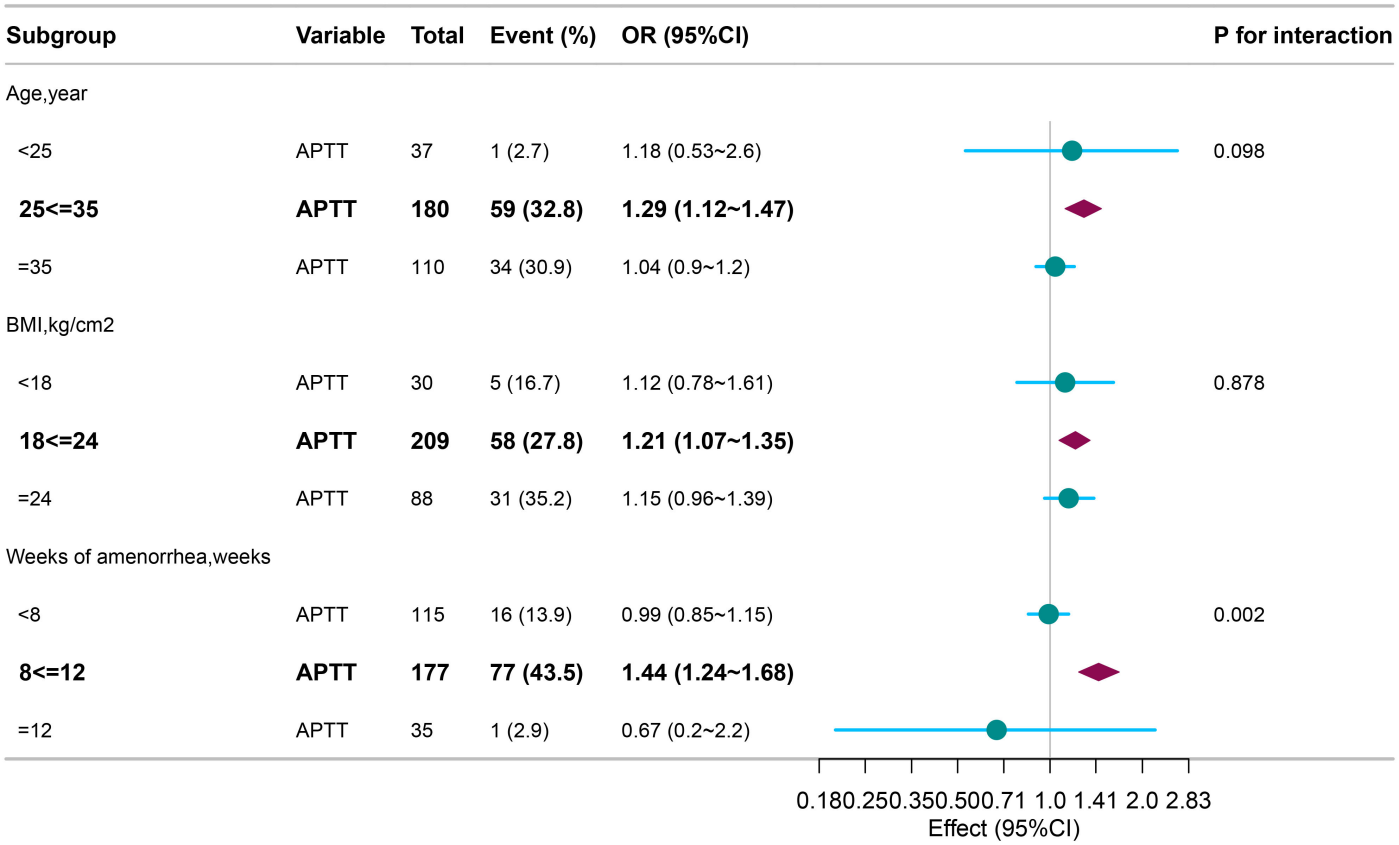
Forest plot showing the interaction effects of APTT with RSA across different subgroups.

**Figure 4:**
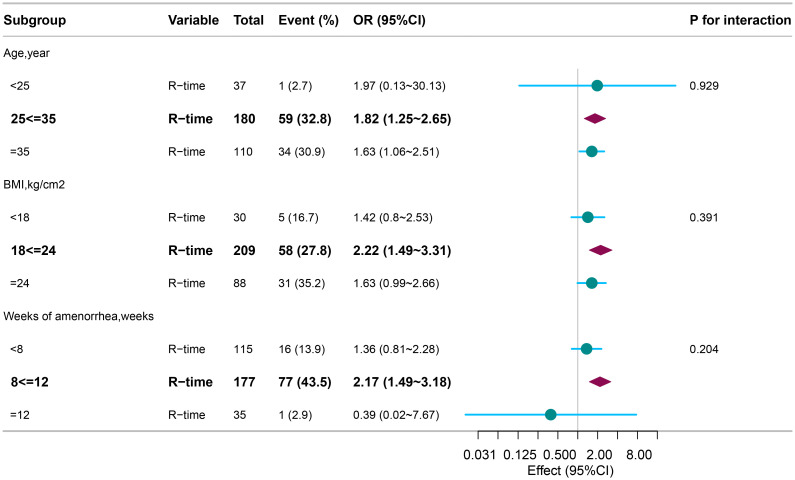
Forest plot showing the interaction effects of R with RSA across different subgroups.

Subgroup analyses (**Table 6**) showed the strongest associations in women aged 25–35 years (APTT OR = 1.29 [1.12-1.47] (**Figure 3**); R-time OR = 1.82 [1.25-2.65]) (**Figure 4**). Significant associations were observed in normal-weight women (BMI 18–24 kg/m²: APTT OR = 1.21 [1.07-1.35]; R-time OR = 2.22 [1.49-3.31]). The most significant associations emerged at 8–12 weeks of amenorrhea (APTT OR = 1.44 [1.24-1.68] (**Figure 3**); R-time OR = 2.17 [1.49-3.18]) (**Figure 4**).

**Table 6:** Subgroup analyses for the association of APTT and R-time with recurrent spontaneous abortion.

Subgroup	Total	Event (%)	APTT	R
			OR (95%CI)	OR (95%CI)
Age(years)				
<25	37	1 (2.7)	1.18 (0.53~2.6)	1.97 (0.13~30.13)
25-35	180	59 (32.8)	1.29 (1.12~1.47)	1.82 (1.25~2.65)
≥35	110	34 (30.9)	1.04 (0.9~1.2)	1.63 (1.06~2.51)
BMI(kg/cm2)				
<18	30	5 (16.7)	1.12 (0.78~1.61)	1.42 (0.8~2.53)
18-24	209	58 (27.8)	1.21 (1.07~1.35)	2.22 (1.49~3.31)
≥24	88	31(35.2)	1.15 (0.96~1.39)	1.63 (0.99~2.66)
Weeks of amenorrhea				
<8	115	16 (13.9)	0.99 (0.85~1.15)	1.36 (0.81~2.28)
8-12	177	77 (43.5)	1.44 (1.24~1.68)	2.17 (1.49~3.18)
≥12	35	1 (2.9)	0.67 (0.2~2.2)	0.39 (0.02~7.67)

### Characteristics of the subjects included normal and missed abortion of CNV-seq negative

Among the 398 patients with missed miscarriage, 250 underwent CNV-seq testing, while 148 declined the test. Comparison between normal controls (n=250) and missed abortion cases with negative CNV-seq results (n=117) revealed no significant differences in age, BMI, days of amenorrhea, D-dimer, TT, PT, FIB, INR, CI, EPL, LY30, missed abortion, angle, or K values (all *P*>0.05). However, the missed abortion group showed significantly prolonged APTT (30.5 ± 2.7 vs. 29.4 ± 2.6 s, *P*<0.001) and R-time (5.1 ± 0.9 vs. 4.9 ± 0.9 min, *P*=0.039) (**Table 7**).

**Table 7:** Coagulation status in women with normal pregnancy and missed abortion with CNV-seq negative results.

Characteristics	Normal(n = 250)	Missed abortion with CNV-seq Negative (n = 117)	*P*-valu*e*
Age (years)	31.8 ± 6.2	31.9 ± 4.2	0.853
BMI (kg/m²)	22.1 ± 3.2	22.5 ± 3.4	0.227
Days of amenorrhea	66.2 ± 24.3	66.6 ± 12.9	0.868
D-dimer(mg/L)	0.3 (0.2, 0.5)	0.3 (0.2, 0.4)	0.671
TT(s)	13.7 ± 1.2	13.8 ± 0.9	0.659
PT(s)	11.4 ± 0.9	11.5 ± 1.2	0.383
APTT(s)	29.4 ± 2.6	30.5 ± 2.7	< 0.001
FIB(g/L)	3.1 ± 0.8	3.4 ± 3.4	0.146
INR	1.1 ± 0.7	1.1 ± 0.1	0.721
CI	1.3 (0.7, 2.2)	1.2 (0.4, 1.9)	0.358
EPL (%)	0.0 (0.0, 1.0)	0.0 (0.0, 0.9)	0.245
LY30 (%)	0.0 (0.0, 0.1)	0.0 (0.0, 0.0)	0.413
MA (mm)	62.9 ± 4.3	63.3 ± 3.8	0.856
Angle(degrees)	67.3 (64.1, 69.5)	68.4 (65.2, 69.7)	0.187
K(min)	1.7 ± 0.9	1.5 ± 0.5	0.204
R-time(min)	4.9 ± 0.9	5.1 ± 0.9	0.039

Data presented as mean ± standard deviation or median (interquartile range) as appropriate. K, Kinetics of clot formation, α-angle; MA, maximum amplitude; LY30, lysis at 30 minutes; EPL, Estimated Percent Lysis; CI, Clotting index. *P* value < 0.05 was considered statistically significant and *P*-value used for each variable is indicated in the corresponding row.

### Subgroup analyses for the association of APTT and R-time with missed abortion with CNV-seq negative

**Figure 5:**
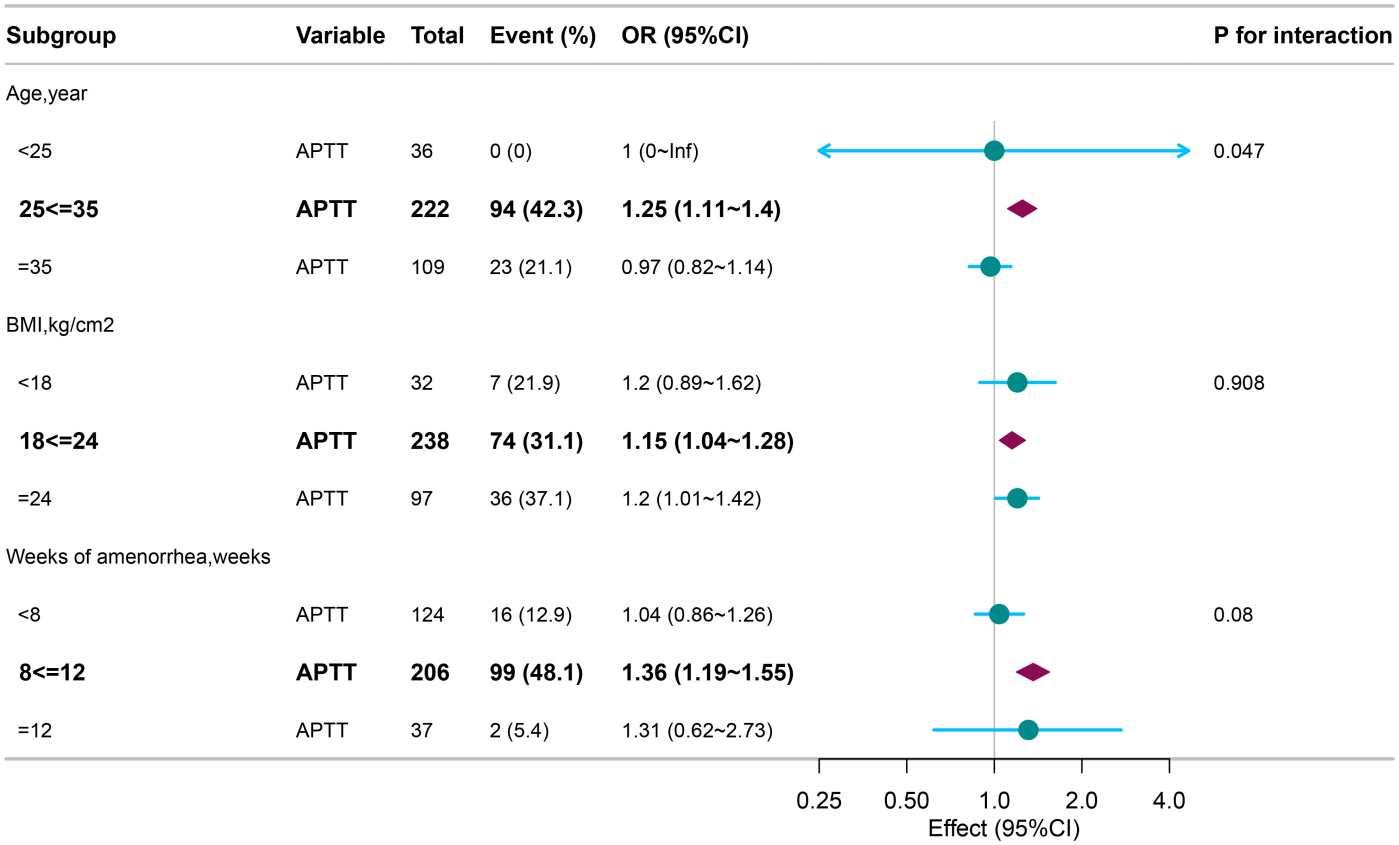
Forest plot showing the interaction effects of APTT between the normal pregnancy and missed abortion of CNV‑seq negative group.

**Figure 6:**
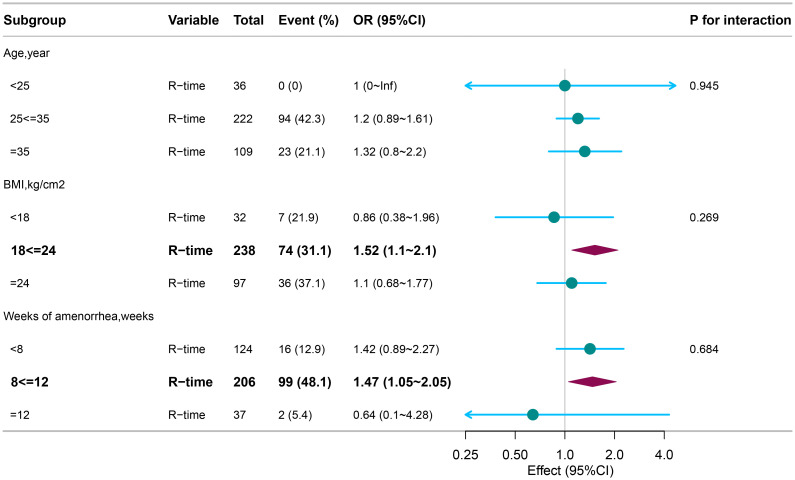
Forest plot showing the interaction effects of R-time between the normal pregnancy and missed abortion of CNV‑seq negative group.

This study evaluated the associations between activated partial thromboplastin time (APTT) and R-time with missed abortion among women with chromosomally normal pregnancies (CNV-seq negative). Subgroup analyses were conducted based on maternal age, BMI, and gestational age. As summarized in **Table 8**, significant associations between APTT and MA were identified in women aged 25–35 years (OR = 1.25, 95% CI: 1.11-1.40) and in those with BMI between 18–24 kg/m² (OR = 1.15, 95% CI: 1.04-1.28) or ≥24 kg/m² (OR = 1.20, 95% CI: 1.01-1.42). Significant associations between R-time and MA were observed in the subgroup with BMI 18–24 kg/m² (OR = 1.52, 95% CI: 1.10-2.10) and in the gestational age subgroup of 8–12 weeks (OR = 1.47, 95% CI: 1.05-2.05). No other subgroups showed significant associations for either parameter. The findings were visualized using forest plots (**Figures 5**, **6**) to illustrate the effect sizes and confidence intervals across these subgroups.

**Table 8:** Subgroup analyses for the association of APTT and R-time with missed abortion in CNV-seq negative cases.

Subgroup	Total	Event (%)	APTT	R
			OR (95%CI)	OR (95%CI)
Age(years)				
<25	36	0 (0)	1 (0~Inf)	1 (0~Inf)
25-35	222	94 (42.3)	1.25 (1.11~1.4)	1.2 (0.89~1.61)
≥35	109	23 (21.1)	0.97 (0.82~1.14)	1.32 (0.8~2.2)
BMI(kg/cm2)				
<18	32	7 (21.9)	1.2 (0.89~1.62)	0.86 (0.38~1.96)
18-24	238	74 (31.1)	1.15 (1.04~1.28)	1.52 (1.1~2.1)
≥24	97	36 (37.1)	1.2 (1.01~1.42)	1.1 (0.68~1.77)
Weeks of amenorrhea				
<8	124	16 (12.9)	1.04 (0.86~1.26)	1.42 (0.89~2.27)
8-12	206	99 (48.1)	1.36 (1.19~1.55)	1.47 (1.05~2.05)
>12	37	2 (5.4)	1.31 (0.62~2.73)	0.64 (0.1~4.28)

### Subject characteristics of MA with positive vs. negative CNV-seq

A comparison of subject characteristics between women with chromosomally abnormal (CNV-seq positive, n=133) and chromosomally normal (CNV-seq negative, n=117) miscarriages revealed no significant differences in maternal age, BMI, days of amenorrhea, or in the majority of coagulation parameters assessed, including D-dimer, TT, PT, APTT, INR, CI, EPL, LY30, MA, and K values (all *P* > 0.05). Minor yet statistically significant differences were observed in fibrinogen (FIB) levels (2.9 [2.7, 3.2] g/L vs. 3.0 [2.8, 3.4] g/L; *P* = 0.005), angle values (65.4 ± 7.6 vs. 67.0 ± 5.1; *P* = 0.048) and MA (63.3 ± 3.8 vs. 61.1± 11.6; *P* = 0.048) (**Table 9**). Notably, APTT and R-time values did not differ significantly between the two groups. These findings indicate that coagulation parameters—specifically APTT and R-time—are comparable in women with cytogenetically normal and abnormal pregnancy loss, suggesting that these measures are not substantially influenced by the chromosomal status of the miscarriage.

**Table 9:** Coagulation status in women with missed abortion: CNV-seq negative vs. positive.

Characteristics	Missed abortion of CNV-seq negative (n = 117)	Missed abortion of CNV-seq positive (n = 133)	*P*-value
Age (years)	31.9 ± 4.2	32.3 ± 4.2	0.401
BMI (kg/m²)	22.5 ± 3.4	23.1 ± 4.0	0.252
Days of amenorrhea	66.6 ± 12.9	67.5 ± 10.5	0.562
D-dimer(mg/L)	0.3 (0.2, 0.4)	0.2 (0.2, 0.4)	0.078
TT(s)	13.8 ± 0.9	13.5 ± 2.1	0.198
PT(s)	11.5 ± 1.2	11.2 ± 2.1	0.179
APTT(s)	30.5 ± 2.7	30.2 ± 4.7	0.643
FIB(g/L)	3.0 (2.8, 3.4)	2.9 (2.7, 3.2)	0.005
INR	1.1 ± 0.1	1.1 ± 0.1	0.902
CI	1.2 (0.4, 1.9)	1.2 (0.2, 2.0)	0.789
EPL (%)	0.0 (0.0, 0.9)	0.0 (0.0, 0.4)	0.126
LY30 (%)	0.0 (0.0, 0.0)	0.0 (0.0, 0.0)	0.079
MA (mm)	63.3 ± 3.8	61.1 ± 11.6	0.048
Angle(degrees)	67.0 ± 5.1	65.4 ± 7.6	0.048
K(min)	1.5 ± 0.5	1.6 ± 0.6	0.195
R-time(min)	5.1 ± 0.9	5.2 ± 1.1	0.301

Data presented as mean ± standard deviation or median (interquartile range) as appropriate. K, Kinetics of clot formation, α-angle; MA, maximum amplitude; LY30, lysis at 30 minutes; EPL, Estimated Percent Lysis; CI, Clotting index. *P* value < 0.05 was considered statistically significant and *P*-value used for each variable is indicated in the corresponding row.

## Discussion

This study found that prolonged APTT and R-time are significantly associated with both missed abortion and recurrent spontaneous abortion, independent of embryonic chromosomal abnormalities. These associations remained stable after adjusting for potential confounders including age, BMI, and days of amenorrhea, and were significant even in CNV-seq negative cases. Our findings suggest that altered coagulation initiation kinetics, potentially reflecting a hypocoagulable state, may represent an independent associated parameter in miscarriage etiology, particularly for missed abortion and recurrent pregnancy loss.

The relationship between APTT and miscarriage risk has been equivocal in previous literature. While empirical evidence indicates that shortened APTT associates with recurrent miscarriage, potentially attributable to elevated factor VIII levels and a prothrombotic state (15, 21), the implications of prolonged APTT remain less clearly defined. However, existing studies have indicated that patients testing positive for anticardiolipin antibodies (ACA) may exhibit prolonged APTT. For example, one study found that 18.8% of ACA-positive patients had prolonged APTT, whereas only 1.6% of ACA-negative patients showed such prolongation (22). Additionally, patients with fetal retention or late intrauterine fetal demise may develop consumptive coagulopathy or overt/occult DIC, which typically prolongs global clotting times (PT/APTT) and lowers fibrinogen and factor activities (23). Besides, some studies have not established significant correlations with miscarriage risk, and its role in specific coagulopathies warrants further investigation (24). Research on R-time and other TEG parameters in pregnancy and miscarriage remains limited. However, previous studies have demonstrated that whole blood coagulation assays can detect abnormal hemostatic profiles not identified by conventional coagulation tests (25). One study applied TEG in pregnant women with complications including missed abortion and fetal death, though some parameters did not show significant differences between normal pregnancy and missed abortion groups (26). Our findings of prolonged rather than shortened APTT associated with pregnancy loss suggest a more complex relationship between coagulation function and miscarriage risk than previously recognized.

Prolonged APTT typically reflects quantitative or qualitative clotting factor deficiencies, the presence of anticoagulants or inhibitors, or autoimmune conditions such as acquired hemophilia A with anti-FVIII antibodies. Nevertheless, we did not perform lupus anticoagulant, anticardiolipin IgG/IgM, or anti-β2 glycoprotein I antibodies, individual clotting factor assays, heparin contamination, or inhuibitor screening in any of participants. APS is a well-established cause of recurrent pregnancy loss and is associated with coagulation abnormalities. It associates with adverse outcomes in critically patients (27) and may result from anticoagulant therapies such as heparin (28). In autoimmune conditions like acquired hemophilia A, autoantibodies against FVIII lead to markedly prolonged APTT and reduced factor activity (29). Our study excluded patients who tested positive for APS. Nevertheless, we did not perform lupus anticoagulant, anticardiolipin IgG/IgM, or anti-β2 glycoprotein I antibody testing in our study population. Antiphospholipid syndrome is a well-established cause of recurrent pregnancy loss and is associated with coagulation abnormalities. Because these tests were not available for our patients, we cannot exclude the possibility that some participants with subclinical or undiagnosed APS were included in the case groups, potentially confounding the observed associations between coagulation markers and pregnancy loss. Inherited disorders such as Factor XI deficiency due to F11 mutations can cause APTT prolongation, often without bleeding manifestations (30, 31). Therefore, our findings should be interpreted as exploratory associations rather than evidence of a mechanistic or etiological link. Thromboelastography has garnered increasing clinical interest for evaluating coagulation function, providing information on factors VIII, IX, XI, and XII activity. The R-time, reflecting intrinsic coagulation pathway initiation, associates with the functionality of these factors. Prolonged R-time may indicate impaired factor activity, potentially contributing to miscarriage pathophysiology. Certain APTT reagents effectively detect mild deficiencies in factors VIII, IX, and XI (32), underscoring TEG’s utility in rapidly assessing coagulation status. However, the same limitation is that we did not perform confirmatory factor assays or inhibitor tests. Therefore, we cannot determine whether the observed APTT or R_time prolongation in missed abortion and RSA groups is attributable to an undiagnosed coagulation factor deficiency, an inhibitor, or other causes such as lupus anticoagulant. Consequently, our findings should be interpreted as an exploratory association rather than a mechanistic or etiological link between APTT prolongation and pregnancy loss.

We considered the possibility that the elevated APTT and R-time values are a consequence rather than a cause of the miscarriage process, for instance, due to consumptive coagulopathy or inflammatory responses following embryonic demise. However, several lines of evidence from our data argue against this being the sole explanation. First, the consistent observation of this trend in women with RSA suggests that this coagulation profile may represent a persistent, underlying maternal trait rather than an acute epiphenomenon of a single pregnancy loss. Second, the association remained significant after stratification by embryonic chromosomal status, indicating that this maternal factor operates independently of fetal genetic quality. While our retrospective design cannot definitively establish causality, these findings strongly posit that a dysregulated coagulation system, as indicated by prolonged APTT/R-time, may be a contributing factor to pregnancy loss and warrants further investigation as a potential risk marker.

This study has several strengths, including a relatively large sample size, comparison across multiple groups, adjustment for key confounders, and combination of traditional coagulation assays with thromboelastography parameters. In our study, the absolute difference in APTT between the missed abortion group and the normal pregnancy group was approximately 1 second. Although this difference reached statistical significance, its clinical significance should be interpreted with caution. Consequently, we do not advocate the use of APTT as a standalone test for the clinical diagnosis or risk stratification of missed abortion. The lack of significant differences in APTT and R-time between CNV-seq-positive and negative groups suggests that maternal coagulation alterations may contribute to miscarriage risk independently of embryonic chromosomal abnormalities, supporting a multifactorial framework integrating genetic, hematologic, immune, and endocrine factors. However, several limitations should be acknowledged. Blood samples were collected after the diagnosis of missed abortion, so we cannot ascertain whether the observed prolongation of APTT and R-time predated the pregnancy loss or occurred as a consequence of trophoblast dysfunction or hormonal changes following embryonic demise. Therefore, our findings should be interpreted as an association rather than a causal relationship. Potential confounders such as antiphospholipid antibodies (including lupus anticoagulant, anticardiolipin antibodies, and anti-β2-glycoprotein I antibodies) and protein C/S were not comprehensively assessed, which may influence APTT and R-time, and thus our results should be interpreted with caution. Interlaboratory variability in testing protocols may affect comparability, and some subgroup analyses were limited by small sample sizes. Furthermore, due to clinical limitations, we were unable to perform CNV-seq on healthy pregnant women with normal pregnancies, which is an additional limitation. Additionally, our study population predominantly comprised patients with a gestational age of 8–12 weeks. Moreover, future studies should include a larger cohort for further analysis where feasible. Finally, the absolute difference in APTT between the missed abortion group and the normal pregnancy group was approximately 1 second. Although this difference was statistically significant (possibly influenced by the relatively large sample size), its clinical significance should be interpreted with caution. Therefore, we do not recommend using APTT alone for clinical diagnosis or risk stratification of missed abortion. If its potential value is to be confirmed, prospective studies combining other coagulation parameters or serial monitoring are warranted. Besides, an additional major limitation is that we did not collect or had substantial missing data on several important clinical confounders, including PCOS, thyroid disorders, uterine anomalies, previous live birth, number of prior miscarriages, and smoking status. Consequently, these variables could not be adjusted for in our regression models. Future prospective studies with systematic data collection on these factors are needed to confirm whether APTT and R-time independently predict pregnancy loss after controlling for these confounders.

## Conclusion

This study provides systematic evidence that prolonged APTT and R-time are independently associated with both missed abortion and RSA, independent of embryonic chromosomal status. These findings highlight coagulation initiation markers as potential candidates for further study. However, prospective cohort studies are required to validate their clinical utility before any application in risk stratification or patient management can be considered. Future research should focus on elucidating the mechanisms underlying these associations, particularly exploring potential hypocoagulable states, subclinical anticoagulant presence, or consumptive processes, and evaluating their potential clinical applications through prospective longitudinal studies.

## Data Availability

The original contributions presented in the study are included in the article/supplementary material. Further inquiries can be directed to the corresponding author.
